# Catheter-Related Bloodstream Infections among patients on maintenance haemodialysis: a cross-sectional study at a tertiary hospital in Ghana

**DOI:** 10.1186/s12879-023-08581-6

**Published:** 2023-10-07

**Authors:** Bismark Opoku-Asare, Vincent Boima, Vincent Jessey Ganu, Elvis Aboagye, Olive Asafu-Adjaye, Anita Ago Asare, Isaac Kyeremateng, Edward Kwakyi, Adwoa Agyei, Eric Sampane-Donkor, Peter Puplampu

**Affiliations:** 1 Department of Medicine, Korle Bu Teaching Hospital, Accra, Ghana; 2 Department of Medicine & Therapeutics, University of Ghana Medical School, Accra, Ghana; 3 West African Centre for Cell Biology of Infectious Pathogens, University of Ghana, Accra, Ghana; 4 Greater Accra Regional Hospital, Accra, Ghana; 5 Department of Community Health, University of Ghana Medical School, Accra, Ghana; 6 Ghana Health Service, Accra, Ghana; 7 Department of Medical Microbiology, University of Ghana Medical School, Accra, Ghana

**Keywords:** Catheter-related bloodstream infections, Central venous catheter, Haemodialysis

## Abstract

**Background:**

Catheter-Related Bloodstream Infections (CRBSIs) are notable complications among patients receiving maintenance haemodialysis. However, data on the prevalence of CRBSIs is lacking. This study was conducted to determine the prevalence and factors associated with CRBSIs among patients receiving haemodialysis in the renal unit of the largest tertiary hospital in Ghana.

**Methods:**

A hospital-based cross-sectional study was conducted on patients receiving maintenance haemodialysis via central venous catheters (CVC) between September 2021 and April 2022. Multivariate analysis using logistic regression was used to determine the risk factors that were predictive of CRBSI. Analysis was performed using SPSS version 23 and a *p*-value<0.05 was statistically significant.

**Results:**

The prevalence of CRBSI was 34.2% (52/152). Of these, more than half of them (53.9%(28/52)) had Possible CRBSI while 11.5% (6/52) had Definite CRBSI. Among the positive cultures, 62% (21/34) were from catheter sites whilst the rest were from peripheral blood. Gram-negative cultures made up 53% (18/34) of positive cultures with the rest being Gram positive cultures. *Acinetobacter baumannii* (33.3% (6/18)) was the commonest organism isolated among Gram-negative cultures whilst *Coagulase negative Staphylococci* (43.7% (7/16)) was the commonest organism isolated among Gram-positve cultures. Gram-negative bacilli were more predominant in this study making up 52.9% of the total bacteria cultured. Sex, duration of maintenance dialysis, underlying cause of End-stage kidney disease, mean corpuscular haemoglobin (MCH), neutrophil count and lymphocyte count were significantly predictive of CRBSI status (*p*<0.05).

**Conclusion:**

There was a high prevalence of CRBSI among patients undergoing haemodialysis. The commonest causative agent was Coagulase negative Staphylococci, however there was a predominance of Gram-negative bacilli as compared to Gram positive cocci. There is a need to set up infection surveillance unit in the renal unit to track CRBSI and put in place measures to reduce these CRBSI.

## Background

End-stage kidney disease (ESKD) which is an advanced stage of chronic kidney disease (CKD) is a common global health challenge that is rapidly increasing the burden and need for renal replacement therapy [1, 2]. The prevalence of CKD in Ghana is reported to be 13.3% [3]. The commonest form of renal replacement therapy (RRT) used to avert the complications of CKD such as uremia is renal haemodialysis [4]. Efficient haemodialysis requires a well-functioning intravascular access which includes a native arteriovenous fistula, an arteriovenous graft or a central venous catheter [5] . The most important risk factor for bacteremia in patients on dialysis according to studies is central venous catheters [6–8]. Catheter related sepsis may either be defined by surveillance or clinically.

The incidence of CRBSI during haemodialysis is high in developed countries such as the United States of America and Canada as well as India, a middle-income country [6, 9–14]. A study in a tertiary referral hospital in South India [6] revealed an incidence rate of 7.34 episodes per 1000 catheter days [7] . High incidence and prevalence of CRBSI have also been reported in countries in Africa and for West Africa, in Nigeria [15, 16]. A laboratory surveillance study in Pretoria, South Africa reported the incidence rate of 10.1 episodes per 1000 catheter days, 3.7 episodes per 1000 admissions and 0.57 per 1000 in-patient days [15].A recent study in Nigeria demonstrated a CRBSI prevalence of 33.3% in patients undergoing haemodialysis [17].

Patients with end-stage renal disease are at increased risk of infection [18]. The risk of CRSI in patients on haemodialysis increases with the length of central venous catheter access dependence [19, 20].

Their increased risk is because of impaired immunity, the presence of comorbidities, malnourishment and the repeated introduction of catheters during haemodialysis which breaks down the natural protective barrier [21].

Potential risk factors for CRBSI include underlying disease (such as lower haemoglobin level, lower serum albumin level, diabetes mellitus, peripheral atherosclerosis), method of catheter insertion, site and duration of catheter insertion [22], poor personal hygiene, occlusive transparent dressing, moisture around the exit-site, *Staphylococcus aureus* nasal colonization, contiguous infections [22], contamination of dialysate or equipment, inadequate water treatment, dialyzer re-use, higher total intravenous iron dose, increased recombinant human erythropoietin dose, and recent hospitalization or surgery [23]. Hospital records from the Korle Bu Teaching hospital (KBTH) which is the third largest hospital in Africa and the leading national referral centre in Ghana suggests increasing frequency of admissions for dialysis patients. In Ghana, there is limited data on CRBSI prevalence and predisposing risk factors although there are reports of increasing cases of ESKD. The study, therefore, sought to determine the prevalence of CRBSIs and associated risk factors among patients on maintenance haemodialysis at the renal unit of KBTH. This study laid bare the magnitude CRBSI had on chronic dialysis programme at the KBTH.

## Methods

### Study setting and design

A hospital-based cross-sectional study was conducted at the renal dialysis unit of KBTH between September 2021 and April 2022. KBTH isthe largest tertiary hospital in Ghana. The renal unit is a subspecialty under the Department of Medicine at the KBTH. There are currently a total of 18 dialysis machines with an isolation area for dialysis of patients who have tested positive for blood borne viruses (HIV, Hepatitis B, C and SARS-COV-2). The unit provides haemodialysis as a form of renal replacement therapy. At the time of study there were about 220 patients receiving chronic haemodialysis and majority of whom received dialysis at least 2 times a week.

### Study population

The study population included all patients aged 18 years and above, diagnosed with ESKD. Those who had been receiving maintenance haemodialysis (MHD) for at least 3 months and had central venous catheters-in-situ for at least 2 weeks were eligible for the study. Patients being treated for alternate sources of infection like pneumonia and malaria, those with AV fistula, those diagnosed with acute kidney injury and those receiving ultrafiltration for heart failure were excluded from the study.

### Operational definitions

The National Kidney Foundation Kidney Disease Outcomes Quality Initiative (KDOQI) [24] definition of CRBSIs was used. The definition is as follows:

#### Definite

Same organism from a semiquantitative culture of the catheter tip (>15 CFU/catheter segment) *and* from a blood culture in a symptomatic patient with no other apparent source of infection.

#### Probable

Defervescence of symptoms after antibiotic therapy with or without removal of the catheter, in the setting in which blood culture confirms infection, but catheter tip does not (or catheter tip does, but blood does not) in a symptomatic patient with no other apparent source of infection.

#### Possible

Defervescence of symptoms after antibiotic treatment or after removal of catheter in the absence of laboratory confirmation of bloodstream infection in a symptomatic patient with no other apparent source of infection.

### Sample size

Using the standard prevalence study formula based on a study in Nigeria [16], the sample size calculated was 234. However, the patients on MHD at the time of study were 220, which was lower than the estimated sample size. Hence using the formula for correcting for a finite population, the estimated sample size calculated with 10% attrition was 130.

### Data collection

**Fig. 1:**
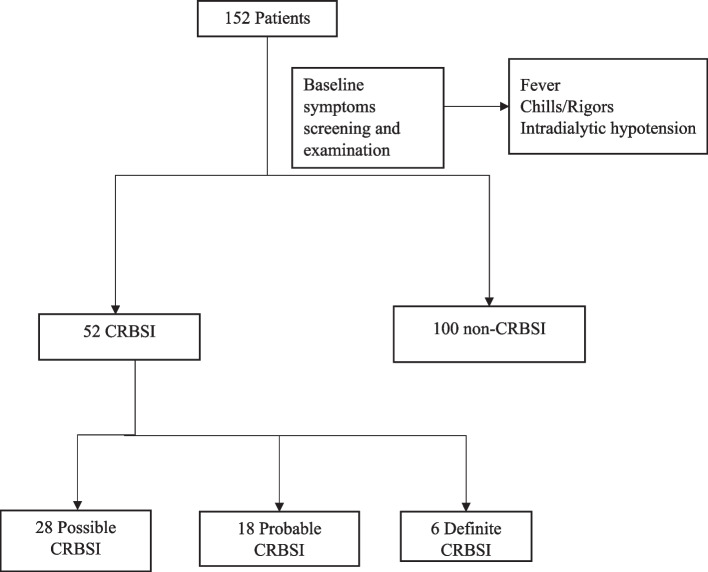
Flowchart for patients recruited into the study

Patients who met the inclusion criteria were recruited after a written informed consent was sought. Recruited patients were screened and examined for CRBSI (Fig. 1).

Trained research assistants administered the study questionnaires whilst two medical officers physically examined the patients diagnosed with CRBSI. Patient information collected included, socio-demographic factors such as the age, sex, marital status, occupation, educational level, the underlying cause of the ESKD, co-morbidities and current medications. Specific details of haemodialysis extracted from the patient medical records included, whether dialysis was initiated as an elective procedure or as an emergency, duration of maintenance haemodialysis, frequency of haemodialysis, catheter insertion site, attendance at out-patient department (OPD) haemodialysis clinic, previous history of CRBSI and the outcomes of the CRBSI treatment. A chest X-ray was done to rule out pneumonia.

#### Sample collection, transportation and culture

A trained laboratory technologist drew 10 mls of whole blood aseptically from both the catheter lumen and the bloodstream from these patients andtransported to the laboratory in an ice chest in blood culture bottles. Blood cultures were processed using automated culture systems (BACTEC 9060) and cultures with a positive signal were sub-cultured by standard methods on Sheep blood agar, chocolate agar and MacConkey agar. The agar plates were incubated overnight, and isolated colonies identified based on colonial morphology, Gram staining and a battery of biochemical reactions, such as the triple sugar iron test, catalase test, urease test, indole test and citrate utilization test were used to identify the bacterial organisms [25].

For patients whose blood cultures were negative (both peripheral or catheter lumen or hub or tip), other alternative diagnoses were ruled out including urinary tract infection, Malaria, chest infection, infective endocarditis, or any abscess collection, and extensive clinical examination was done to rule out any other sources of infection. After ruling out alternative diagnoses, then the most likely source was determined to be the catheter.

### Statistical analysis

All data were entered in Microsoft Excel 2016. The data was exported and analyzed using SPSS version 23. Percentages were computed to present variables such as age, sex, causative microorganisms. The chi-square test was performed to compare demographic and clinical variables (age, sex, comorbidities, duration of maintenance haemodialysis, central venous catheter (CVC)insertion site) between the groups of patients with and without CRBSI. The *p*-value of less than 0.05 was considered statistically significant. Multivariate analysis using logistic regression was used to determine the risk factors that are predictive of CRBSI.

## Results

### Background characteristics of study participants

A total of 152 patients undergoing maintenance haemodialysis were recruited and screened for CRBSI. Of the number screened, 34.2% (52/152) had CRBSI. Of those who had CRBSI, their mean age was 45.2±14.3. Majority (61.5% (32/52))were male. Most (44.2% (23/52)) had at least secondary form of formal education (Table 1). The commonest underlying cause of ESKD were hypertension (48.1% (25/52)); retroviral infection (21.1% (11/52); and diabetes (15.4% (8/52)) Eighty seven percent (45/52) of patients had dialysis initiated as an emergency and 73.1% (38/52)had central venous catheters inserted through the right internal jugular vein. (Table 1)

**Table 1:** Background characteristics of study participants with CRBSI at the Korle Bu Teaching Hospital, Accra, Ghana, 2021-2022

**Characteristic**	**Frequency**	**Percentage**
**Age**		
Mean ± SD	45.2 ± 14.3	
< 25	3	5.8
25-49	35	67.3
50-59	8	15.4
≥60	6	11.5
**Sex**		
Female	20	38.5
Male	32	61.5
**Marital status**		
Divorced	1	1.9
Married	31	59.6
Single	17	32.7
Widowed	3	5.8
**Education level**		
Functionally illiterate	3	5.8
Primary	10	19.2
Secondary	23	44.2
Tertiary	16	30.8
**Underlying cause of ESKD**		
Chronic Glomerulonephritis	3	5.8
Diabetes	8	15.4
Hypertension	25	48.1
Retroviral infection	11	21.1
Others^a^	5	9.6
**Haemodialysis initiation**		
Emergency	45	86.5
Elective	7	13.5
**Duration of maintenance dialysis**		
3- <6months	39	74.9
6- <12months	7	13.5
1-5years	4	7.7
>5years	2	3.9
**Frequency of haemodialysis**		
Once a week	8	15.4
Once or twice a week	1	1.9
Twice a week	39	75
Three times a week	4	7.7
**Catheter insertion site**		
Left femoral vein	1	1.9
Right femoral vein	13	25.0
Right internal jugular vein	38	73.1
**Regular dialysis clinic attendance**		
No	13	25
Yes	39	75
**Previous history of CRBSI**		
No	43	82.7
Yes	9	17.3

^a^Alport syndrome, SLE, Polycystic kidney disease, Lupus nephritis; *ESKD* End-stage kidney disease, *CRBSI* Catheter-related bloodstream infections, *SD* Standard deviation

### Prevalence of CRBSIs

**Fig. 2:**
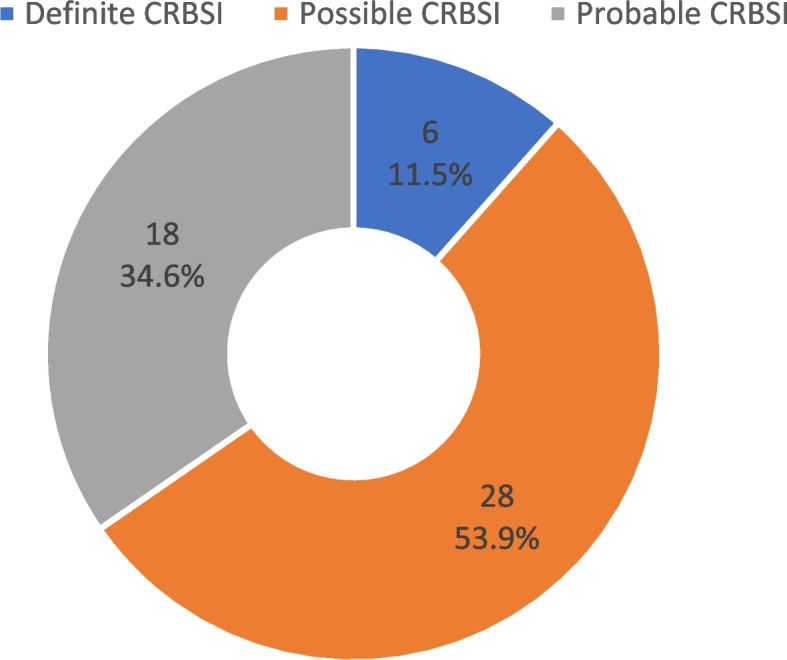
Classification of CRBSI among study participants at the Korle Bu Teaching Hospital, Accra, Ghana, 2021-2022.

The prevalence of CRBSI was 34.2% (95% CI: 26.7 – 42.3%). Among patients with CSRBI, more than half (53.9%(28/52))) had Possible CRBSI while 11.5% (6/52) had Definite CRBSI (Fig. 2).

Majority of the patients presented with general malaise(77%), fever(73%),chills/rigors(67.3%), and vomiting(44.2%). (Table 2).

**Table 2:** Clinical symptoms amongst study participants with CRBSI at the Korle Bu Teaching Hospital, Accra, Ghana, 2021-2022

	**Frequency**	**Percentage**
General malaise	40	77.0
Fever	38	73.0
Chills/Rigors	35	67.3
Vomiting	23	44.2
Nausea	17	32.7
Altered mental status	13	25.0
Intradialytic hypotension	16	30.7
Catheter dysfunction	7	13.5
Hypothermia	1	0.2

### Physical examination

Weight and height could not be measured for 15 participants because they were neither able to ambulate nor stand. Hence no body mass index was computed for these patients. Among the 137 participants with BMI, 38.0% had normal BMI; 34.3% were overweight. Almost all (94.7%) participants were clinically pale. About half (52%) had pedal oedema.

### Predictors of CRBSIs among study participants

The multivariate logistic regression model showed that sex, duration of maintenance dialysis, underlying cause of ESKD, mean corpuscular haemoglobin (MCH), neutrophil count and lymphocyte count were significantly predictive of CRBSI status (*p*<0.05). From the adjusted logistic regression model, the odds of having CRBSI was about 6 times higher among males compared to females (aOR: 5.74, 95%CI:1.24 -26.55). The odds of developing CRBSI were 78% lower among participants whose ESKD was caused by diabetes compared to those whose ESKD was caused by other causes (aOR: 0.22, 95%CI: 0.05 – 0.93). After adjusting for all other co-variates, the odds of having CRBSI was 93% lower among participants who have been on maintenance dialysis for a year or more compared those who have been on maintenance dialysis for less than a year (aOR: 0.07, 95%CI: 0.01 – 0.62). (Table 3)

**Table 3:** Predictors of CRBSI of study participants at the Korle Bu Teaching Hospital, Accra, Ghana, 2021-2022

	**Unadjusted Logistic Regression Model**			**Adjusted Logistic Regression Model**		
	**uOR**	**95% CI**	***P*-value**	**aOR**	**95% CI**	***P*-value**
**Age**	1.01	0.99 – 1.03	0.495	1.05	0.99 – 1.11	0.078
**Sex**						
Female	1.00			1.00		
Male	1.48	0.74 – 2.93	0.264	5.74	1.24 – 26.55	**0.025**
**Haemodialysis initiation**						
Emergency	1.00			1.00		
Elective	0.55	0.22 – 1.4	0.209	0.28	0.04 – 2.14	0.221
**Underlying cause of ESKD**						
Others	1.00			1.00		
Hypertension	0.55	0.2 – 1.51	0.244	0.30	0.03 – 2.76	0.288
Diabetes	0.63	0.3 – 1.36	0.24	0.22	0.05 – 0.93	**0.04**
**Duration of MHD**						
<1year	1.00			1.00		
≥1year	0.34	0.13 – 0.88	0.026	0.07	0.01 – 0.62	**0.017**
**Frequency of MHD**						
<3times	1.00			1.00		
≥3times	0.61	0.19 – 2.01	0.417	0.24	0.02 – 2.77	0.254
**Catheter insertion site**						
Femoral Vein	1.00			1.00		
Jugular Vein	1.05	0.49 – 2.28	0.894	0.89	0.15 – 5.26	0.896
**Regular clinic follow-up**						
No	1.00			1.00		
Yes	1.05	0.49 – 2.28	0.894	0.66	0.14 – 3.12	0.604
**Previous CRBSI**						
No	1.00			1.00		
Yes	1.53	0.6 – 3.93	0.372	6.61	0.57 – 76.97	0.132
**Hb(g/dl)**						
<10 Low	Low			1.00		
10-12 Normal	0.45	0.12 – 1.67	0.232	0.22	0.01 – 3.76	0.296
>12 High	0.90	0.08 – 10.22	0.929	1.29	0.05 – 35.86	0.879
**MCV (fL)**						
<75 Low	1.00			1.00		
75-100 Normal	0.80	0.33 – 1.92	0.617	1.96	0.28 – 13.82	0.498
**MCH (pg)**						
<25 Low	1.00			1.00		
25-30 Normal	0.59	0.28 – 1.23	0.16	0.20	0.03 – 1.49	0.117
>30 High	0.49	0.11 – 2.12	0.343	0.07	0.01 – 0.41	**0.003**
**Neutrophil count (x 10**^**9**^**/L)**						
<2 Low	1.00			1.00		
2-7 Normal	0.38	0.08 – 1.72	0.207	0.01	0.001 – 0.12	**0.001**
>7 High	2.47	0.57 – 10.75	0.229	0.71	0.01 – 34.85	0.865
**Lymphocyte count (x 10**^**9**^**/L)**						
<1 Low	1.00			1.00		
1-3 Normal	1.87	0.69 – 5.05	0.22	11.29	1.62 – 78.64	**0.014**
>3 High	1.19	0.24 – 5.99	0.835	0.85	0.07 – 10.73	0.901
**Platelet count (x 10**^**9**^**/L)**						
<150 Low	1.00			1.00		
150-400 Normal	0.59	0.26 – 1.37	0.219	0.43	0.09 – 2.14	0.301
>400 High	1.34	0.45 – 3.96	0.597	2.58	0.35 – 19.07	0.353

*uOR* Unadjusted odds ratio, *aOR *Adjusted odds ratio, *CI *Confidence interval, *HD* means haemodialysis, *ESKD* means end-stage kidney disease, *CRBSI* means Catheter-related bloodstream infection, *Hb* means haemoglobin, *MCV* means mean corpuscular volume, *MCH* means mean corpuscular haemoglobin, *g/dl* gram per decilitre, *fL* Femtolitre, pg: picogram, *L* Litre

### Microbial causative agents

Of the 104 cultures that were conducted for patients with CRBSI, the culture positivity rate was 32.7% (34/104). For cultures that were positive, 62% (21/34) were from catheter site and the rest were from peripheral blood cultures. Of all positive cultures, 47.1% (16/34) yielded Gram-positive organisms and 52.9% (18/34) yielded Gram-negative organisms. *Coagulase negative Staphylococci* (43.7%) was the commonest cultured Gram-positive organism, followed by *Staphylococcus aureus* (31.3%) (Table 4). Among the Gram-negative organisms cultured, *Acinetobacter baumannii* (33.3%), *Pseudomonas aeruginosa* (22.2%) and *Klebsiella pneumoniae* (22.2%) were the most cultured organisms. (Table 4)

**Table 4:** Microbial organisms cultured among blood cultures conducted among patients with CRBSI at the Korle Bu Teaching Hospital, Accra, Ghana, 2021-2022

**Gram Positive Organisms (*n*=16)**		**Gram Negative Organisms (*n*=18)**	
	N (%)		N (%)
*Coagulase Negative Staphylococci*	7(43.7)	*Acinetobacter baumannii*	6(33.3)
*Staphylococcus aureus*	5(31.3)	*Pseudomonas aeruginosa*	4(22.2)
*Enterococcus*	2(12.5)	*Klebsiella pneumoniae*	4(22.2)
*Bacillus (Most likely contaminant)*	2(12.5)	*Escherichia coli*	2(11.1)
		*Enterobacter*	1(5.6)
		*Citrobacter*	1(5.6)

## Discussion

The prevalence of catheter-related bloodstream infections (CRBSI) was 34.2% among the study participants. Among the 52 participants with CRBSI, more than half of them had possible CRBSI, 34.9% had probable CRBSI and the rest had definite CRBSI. General malaise, fever and chills or rigor were the most common clinical presentation. Risk factors that were independently predictive of CRBSI included male sex, duration of maintenance dialysis, the underlying cause of ESKD (diabetes), MCH, neutrophil and lymphocyte count.

The prevalence of CRBSI among patients on haemodialysis in KBTH according to this study is 34.2%. Other centres globally have reported lower rates of between 4.2 and 5.6% [26, 27]. The prevalence in this study however is comparable to a study done in Nigeria which reported a prevalence of 33.3% amongst 171 patients on haemodialysis [28]. A previous study in Nigeria reported a much lower prevalence of 18.8% and this was attributed to a majority of the patients being lost to follow up and it may therefore have been an under-representation of what actually pertains [16]. There are several reasons that could account for the high prevalence of CRBSI in our renal unit. Currently at the unit there is no written down protocol with regards to catheter care and infection prevention. Skin asepsis before the insertion of CVC is done with povidone iodine and 70% isopropyl alcohol but the standard of care however is use 2% aqueous chlorhexidine glucuronate and 70% isopropyl alcohol [29]. The use of 2% aqueous Chlorhexidine glucuronate plus 70% isopropyl alcohol has been shown to significantly inhibit growth of normal skin as compared to those with 10% povidone iodine plus 70% isopropyl alcohol [30].

The high prevalence of CRBSI will therefore require a more stringent application of all the preventive strategies required like enforcing all patients use of topical antimicrobials like mupirocin in the unit and strict enforcement of all infection prevention control (IPC) measures by staff and patients alike. Another contribution to the increased prevalence of CRBSI as reported by this study may partly be the result of the non-use of the antiseptic/antibiotic lock system. It has been shown that the prophylactic use of a combination of an antibiotic-anticoagulant catheter lock system results in a 50-100% reduction in blood stream infections [31]. The Centre for Disease Control Dialysis Collaborative published a list of comprehensive dialysis management protocol in 2011 [32]. A study was carried out with this protocol in 17 outpatient dialysis units which reporting findings of a 54% reduction (*P*<0.001) in catheter related blood stream infections [29]. Nasal Staphylococcus aureus decolonisation has been shown to reduce the risk of CRBSI [33]. A study done in the northern region of Ghana demonstrated high Staphylococcus aureus nasal carriage among health care workers, in patients and caregivers with health care workers having the highest rate of Methicillin-resistant Staphylococcus aureus (MRSA) carriage [34]. Further studies to determine MRSA carriage among health care workers in our setting is paramount to determine its contribution to CRBSI.

From our study, the odds of having CRBSI was about 6 times higher among males compared to females. However, this effect should be treated with caution due to its wider confidence interval. Studies elsewhere have reported mixed results with regards to gender. A study by Hadian et al involving 122 patients on haemodialysis reported that male gender was a statistically significant risk factor for the development of CRBSI [35]. Another study by Gomez et al reported that more males developed CRBSI compared to females [27]. A study by Fysaraki et al also found that females had more CRBSI as compared to males [21] but a study by Mohamed et al however demonstrated no gender effect on infection rates [36]. Age in this study was not found to be significantly associated with CRBSI but a study conducted by Murea et al demonstrated that the elderly are at a lower risk of catheter related blood stream infections in dialysis compared to their younger counterparts [28].

This study found also out that odds of developing CRBSI was 78% lower among participants whose ESKD was caused by diabetes compared to those whose ESKD was caused by other causes. The study in addition found out that the second most common cause of ESKD was diabetes (18.4%) and hypertension was the most common cause of ESKD making up 52% of the cases seen. In most developing countries though, the leading cause of ESKD [37] is diabetes and presently the leading cause of ESKD in Sub Saharan Africa is hypertension but diabetes is fast becoming the leading cause [38, 39]. The few number of patients with diabetes in this study may have affected the outcomes in this study and more work needs to be done in another study with a larger sample size to determine the true effect of diabetes mellitus on the prevalence of CRBSI.

In our study, the culture positivity rate among those who had CRBSI was 32.7%. This culture positivity was similar to findings from another study by Bello et al reporting a culture positivity among patients with CRBSI of 33.3% [17] but higher than findings of culture positivity of 27.3% by Amira et al [16] .. However, a study by Farrington et al reported an culture positivity of 85% [19] which was higher than the findings in our study. This higher culture positivity rate could be as a result of well enforced restrictions on use of antibiotics in their populations and also the ability to culture fastidious organisms in most of these centres [40] which is lacking in developing countries such as Ghana [41].

Most patients after reporting to peripheral clinics in Ghana may have been exposed to antibiotics before a referral to the teaching hospital. Hence, there is a reduced likelihood of culturing any organism in their blood at the tertiary level Another reason for the lower culture positivity rate in our study may be that only aerobic cultures were done in this study and therefore the possibility of missing out on diagnosis of fastidious bacteria which will therefore result in under-reporting of positive cultures. In developed countries, the picture is quite different as they report much higher culture positivity.

Notably, a little less than half (47.1%) of the bacteria cultured was Gram-positive and 52.9% of the organisms cultured were Gram negative indicating a predominance of Gram-negative bacteria. This is in keeping with recent global studies which have reported a shift in the epidemiology of CRBSI from Gram positives to Gram negatives [42–44]. It has been suggested by El-Kady et al that in health settings, a high rate of infection with Gram negative bacilli should raise concern about possible inadequate hand hygiene and poor compliance to catheter maintenance precautions [44]. The most common Gram-positive bacteria cultured in this study however was *coagulase negative Staphylococci* (20.6%) This finding is similar to other studies where Coagulase negative Staphylococci was also reported asthe most predominant Gram-positive bacterial causative agent [36, 44].

### Study limitations

Anaerobic cultures were not conducted in this study which is limitation. Our non-conduct of anaerobic cultures, which identifies fastidious bacteria, may have led to under-reporting of culture positivity in our study.

## Conclusion

There is high prevalence (34.2%) of CRBSI at the KBTH dialysis unit. The factors significantly predictive of CRBSI among patients include male sex, short duration of maintenance dialysis using the CVCs, ESRD caused by diabetes, neutrophil count, and lymphocyte count. More extensive work needs to be done to study CRBSI in more patients over a longer period and in other renal haemodialysis units all over the country. This will give us more encompassing data to guide the prevention and treatment of CRBSI and reduce the complications associated with CRBSI.

## Data Availability

The datasets generated and/or analysed during the current study are not publicly available due the regulations of the KBTH Ethics Committee and IRB regulations but are available from the corresponding author (Dr Peter Puplampu, pedpup2@gmail.com) on reasonable request.

## Abbreviations

CRBSIs: Catheter-Related Bloodstream Infections
ESKD: End-stage kidney disease
MHC: mean corpuscular haemoglobin
CKD: chronic kidney disease
RRT: renal replacement therapy
KBTH: Korle Bu Teaching hospital
HIV: Human Immunodeficiency Virus
KDOQI: Kidney Disease Outcomes Quality Initiative
MHD: Maintenance Haemodialysis
OPD: out-patient department
PI: Principal Investigator
